# Pediatric Pain Management: An Observational Study on Nurses’ Knowledge of Non-Pharmacological Techniques

**DOI:** 10.3390/nursrep15080290

**Published:** 2025-08-09

**Authors:** Lum Jusufi, Enrico Cocchi, Rita Blaco, Valeria Cremonini, Claudia Cadas, Elsa Vitale, Roberto Lupo, Giorgio De Nunzio, Donato Cascio, Gianandrea Pasquinelli, Luana Conte, Ivan Rubbi

**Affiliations:** 1 Azienda USL Romagna, Ospedale di Ravenna, 48121 Ravenna, Italy; lum.jusufi@auslromagna.it (L.J.); rita.blaco@auslromagna.it (R.B.); 2 Dipartimento di Scienze Mediche e Chirurgiche, University of Bologna, 40126 Bologna, Italy; enrico.cocchi3@unibo.it (E.C.); gianandr.pasquinelli@unibo.it (G.P.); 3 School of Nursing, Campus of Ravenna-Faenza, University of Bologna, 48018 Faenza, Italy; valeria.cremonini@auslromagna.it (V.C.); claudia.cadas@auslromagna.it (C.C.); ivan.rubbi2@unibo.it (I.R.); 4 Directorate of Health and Nursing Professions, Local Health Authority (ASL) of Bari, 70123 Bari, Italy; elsa.vitale@asl.bari.it; 5 San Giuseppe da Copertino Hospital, Local Health Authority (ASL) of Lecce, 73043 Copertino, Italy; roberto.lupo@unisalento.it; 6 Laboratory of Biomedical Physics and Environment, Department of Mathematics and Physics “E. De Giorgi”, University of Salento, 73100 Lecce, Italy; giorgio.denunzio@unisalento.it; 7 Laboratory of Data Analysis for Medicine (ADAM), University of Salento and Local Health Authority (ASL) of Lecce, 73100 Lecce, Italy; 8 Department of Physics and Chemistry ‘E. Segrè’, University of Palermo, 90128 Palermo, Italy; donato.cascio@unipa.it

**Keywords:** pediatric pain management, non-pharmacological techniques, nursing practices

## Abstract

**Introduction**: Pain represents a significant threat to the physical and psychological well-being of children, negatively affecting their quality of life during hospitalization. Pain is considered the fifth vital sign and must be regularly assessed and managed, as also emphasized by the nursing code of ethics. The interdisciplinary approach to pediatric pain management includes both pharmacological treatments and non-pharmacological techniques (NPTs), taking into account the child’s age and specific needs. NPTs comprise a broad set of methods, ranging from simple to complex, that can be applied to children to help them manage pain. The main objective of this study was to explore and analyze which non-pharmacological methods are adopted by nurses in their clinical practice to relieve pain in school-aged children (6–12 years) undergoing surgery. **Materials and Methods**: This observational study involved nursing staff from pediatric wards in the Italian provinces of Ravenna, Forlì-Cesena, and Rimini, and used a validated online questionnaire. The study focused on school-aged children (6–12 years) who had undergone surgical procedures. The questionnaire included items on which NPTs nurses used to relieve pain in pediatric patients. Participants responded using a Likert scale from 1 (never) to 5 (always), and anonymity and voluntary participation were guaranteed. Data were collected between February and October 2024, involving the pediatric units of three hospitals in the provinces of Ravenna, Forlì-Cesena, and Rimini. Statistical analyses included *t*-tests, ANOVA, and Kruskal–Wallis tests to identify significant differences. **Results**: A total of 46 nurses completed the questionnaire. No significant differences were found between nurses’ backgrounds and the use of NPTs. Overall, nurses did report using NPTs, although there was limited use of such techniques in the preoperative phase. The study also highlighted a discrepancy in the information provided to children versus parents, with nurses tending to give more information to parents during the preoperative period. Notably, nurses who reported effective multidisciplinary collaboration were also those who better prepared children using NPTs. **Conclusions**: This study emphasizes the importance of NPTs in pediatric pain management and highlights the need to improve direct communication with children. Adopting an effective multidisciplinary approach is essential to ensuring a less traumatic surgical experience for young patients.

## 1. Introduction

Among all symptoms, pain is the one that most threatens the physical and psychological integrity of children and causes great concern for their families, significantly impacting their quality of life during hospitalization and beyond. Pain is considered the fifth vital sign and, as such, must be regularly assessed in all patients [1].

Pain assessment in children should take into account their age, cognitive level, any disabilities, the type of pain, and the context in which it occurs. The recommended interdisciplinary therapeutic approach to pediatric pain management includes both pharmacological and non-pharmacological techniques. The latter are highly varied but can be grouped into two main categories: cognitive-behavioral methods and physical methods [2,3].

To accurately assess pain in children, it is essential to consider what the patient reports, their behavior, and their physiological responses. Pain scales are used to provide an objective measure of the subjective experience of pain. Various types exist, such as the FLACC scale and the Wong–Baker scale, as well as multidimensional assessment tools like PROMIS^®^ (Patient-Reported Outcomes Measurement Information System) [4].

Pediatric patients often experience moderate to severe pain following surgical procedures in the hospital. When acute pain persists during the postoperative period, it may evolve into chronic pain, also known as chronic postsurgical pain (CPSP). This transition from acute to chronic pain in children can be described through a model in which psychological, social, and behavioral factors—related to the child’s age, sex, and genetic profile—modulate changes in sensory processing in response to pain, potentially leading to chronic pain [5]. Several studies in the literature present a concerning picture: healthcare professionals often have limited knowledge regarding pediatric pain management [6,7,8]. In one study, it was even shown that only two-thirds (67.5%) of nurses were competent in pain management [9].

Pain is a complex sensory modality, a system that enables interaction with the external environment and is essential for survival [10]. It can negatively impact a child’s development and overall quality of life. Acute pain has been shown to be associated with distress and anxiety, while chronic pain is linked to feelings of helplessness and depression. Painful medical procedures are among the primary sources of distress in pediatric patients and can have long-term consequences on behavior, memory, pain perception, and developmental outcomes [11]. Pain management is often suboptimal due in part to the limited amount of pediatrics-specific research and concerns about the side effects of certain analgesics. An updated WHO scale addressing acute postoperative pain supports several recommendations for proper analgesic use: favoring the oral route (when possible); administering analgesics at regular intervals; dosing according to pain severity; tailoring doses to individual patients; and closely monitoring pharmacological prescriptions [12]. To alleviate pain in pediatric patients, nurses can adopt non-pharmacological techniques (NPTs), which include a broad set of strategies, methods, and tools—ranging from simple to complex—that can be applied to children to help them control pain. These techniques can be grouped into the following categories: supportive/relational, cognitive-behavioral, and physical. Children naturally possess a vivid imagination that is far more developed than that of adults. NPTs are based on this innate potential, helping children focus on positive or distracting imagery rather than the tension, pain, or discomfort they are experiencing.

Trust between healthcare professionals and patients is primarily established through eye contact and physical touch, which lay the foundation for a therapeutic relationship. Several studies have demonstrated that touch and massage can have a significant impact on the neurophysiological response, promoting the release of chemical mediators that activate the vagus nerve and reduce stress and pain. Eye contact activates mirror neurons, and the interaction between two individuals triggers the release of oxytocin and vasopressin, which help reduce stress. This chemical process increases the level of trust and empathy between those involved [13].

The key feature of cognitive-behavioral techniques is the active involvement of the child, which fosters a sense of control over what is happening. Clown therapy is a method used to enhance pediatric patients’ well-being by employing humor and evoking positive emotions. In a study by Markova et al. [14], the authors investigated the positive effects (such as reduced negative emotions and increased positive ones) of hospital clowns on pediatric patients in the preoperative phase. The study involved 62 school-aged children (5–12 years) who were randomly assigned to an intervention group or a control group. The Modified Yale Preoperative Anxiety Scale (mYPAS), along with other tools, was used for evaluation. The results showed that children in the intervention group reported a more positive experience compared to those in the control group [14]. A prospective observational comparison study was conducted in a burn outpatient clinic that operated twice a week. It compared sessions with the presence of a medical clown once a week to sessions without a clown. Patients and accompanying persons filled out pain (WBS and VAS) and emotional distress (SUDS) questionnaires regarding the patient’s experience, before (T1) and after treatment (T2). Clinic staff also filled out SUDS questionnaires at the start and end of the clinic’s working hours. The results showed significantly lower scores for WBS, VAS, and SUDS at T2 in the experimental group (EG) compared to the control group (NEG), as reported by both patients and their companions. Similarly, the SUDS scores of the clinic personnel were also affected in the same way. The presence of the medical clown induced a positive atmosphere in the clinic, which was reflected in the reduction in pain parameters and distress scores [15].

Physical interventions aim to alter the sensory dimension of pain by modifying the reception of nerve impulses or activating endogenous mechanisms of pain suppression. Transcutaneous electrical nerve stimulation (TENS) has been widely used to treat both acute and chronic pain. It is effective in 67% of different types of pain and is a device that releases continuous or intermittent vibrations through electrodes placed on the skin. It is simple to use, even with young patients [16]. The study of pain and nociception is a broad and multifaceted field that requires an approach recognizing the interaction of multiple systems [17].

The main objective of this research was to explore and analyze which non-pharmacological methods are adopted by nurses in their clinical practice to relieve pain in school-aged children (6–12 years) undergoing surgery. The study also aimed to investigate how background factors (sociodemographic context) may influence the use of non-pharmacological strategies in managing pediatric pain. An additional outcome of the research was to analyze the relationship between interventions aimed at children and those aimed at parents. Analyzing these factors could provide valuable insights for improving nursing care in pediatric pain management. To achieve these objectives, an observational study was conducted.

## 2. Methods

Study Design: This was a multicenter observational study.

Setting: The study was conducted between February and October 2024. The questionnaire was distributed via a link to the nursing coordinators of the pediatric departments. The coordinators took care to share the link with the nurses; the link redirected the voluntary participants to the Microsoft Forms platform. Once responses were submitted by the participants who had been recruited on a voluntary basis, the data were automatically recorded into a database.

Nurses who agreed to participate in the study could complete the questionnaire via self-administration. An introductory email presenting the study was sent to the coordinators of the relevant hospital units, who were asked to share the invitation with their nursing staff.

Participants: A non-probabilistic (convenience) sample was used. The study involved nurses working in pediatric hospital settings within a healthcare organization in the provinces of Ravenna, Forlì-Cesena, and Rimini. The voluntary sample was recruited via email, which was sent to the coordinators of the hospital units.

All healthcare professionals working in pediatric wards and providing direct patient care were included in the study. Nurses with exclusively organizational and coordination roles were excluded.

Instrument: The data collection tool was the questionnaire developed by Pölkki et al. [18], with prior authorization from the author. The validity of the questionnaire was confirmed by administering it to a sample of 162 nurses working in pediatric surgery departments in five Finnish university hospitals. The age, education, and work experience of the nurses showed a significant correlation with the use of non-pharmacological methods. More experienced nurses and those with children tended to use such methods more frequently. The study has shown that greater training and support are needed for new nurses to improve pain management in children. The questionnaire was divided into several sections. The first section explored sociodemographic characteristics, adapted to the Italian context. The subsequent sections collected information on the non-pharmacological techniques (NPTs) used by nurses for school-aged children experiencing pain in the preoperative and postoperative periods. These areas included the following variables and quantitative items: (a) cognitive-behavioral methods (such as preparatory information, imagery, distraction, relaxation, breathing techniques, and positive reinforcement—item 42); (b) physical methods (such as thermal regulation, massage, positioning, and transcutaneous electrical nerve stimulation—item 5); (c) emotional support (including presence, comforting/reassurance, and touch—item 3); (d) assistance with daily activities (item 1); and (e) Creating a comfortable environment (item 6).

A final section focused on interventions addressed to parents: (f) parental guidance. Nurses responded to affirmatively worded statements regarding the use of non-pharmacological techniques, using a five-point Likert scale (1–5), ranging from “not at all” and “very seldom” to “sometimes”, “nearly always”, and “always”. The questionnaire also included open-ended questions under the item “other”, allowing participants to freely add additional comments. An additional section with six descriptive questions was appended to the instrument, the questions were added by nursing staff with expertise and experiences in pediatrics care, each using a four-point Likert scale (1–4), ranging from “not at all” and “a little” to “quite a bit” and “very much” for a total of 59 questions.

Statistical Analysis: Statistical analyses were conducted using Jamovi for Windows (version 8.0). Descriptive statistics were used to summarize the nurses’ demographic characteristics and their use of non-pharmacological methods. Composite variables were created from those that were interrelated (based on inter-item correlations and Cronbach’s alpha) and conceptually connected. The sample size was determined using the Kaiser–Meyer–Olkin (KMO) measure.

The statistical association between background factors and all non-pharmacological methods was assessed using the chi-square test. For this analysis, the six dimensions representing the use of pain relief methods were grouped into three categories: “never/very rarely/sometimes” and “almost always/always” [18].

The statistical significance of the differences between the mean scores of the instrument’s dimensions and the sample variables was tested using *t*-tests and ANOVA. Multiple comparisons were performed using Tukey’s HSD test. A *p*-value < 0.05 was considered statistically significant.

### Ethical Considerations

Before participating in the study, nurses received clear information about the study’s objectives, the voluntary nature of participation, potential risks and benefits, and how their personal data would be handled. A detailed explanation of the study protocol was provided to enable informed decision-making. Participation was entirely voluntary, and nurses had the right to withdraw from the study at any time without any negative consequences. This policy was essential to ensure that only those who felt comfortable sharing their experiences took part in the research.

The study received ethical approval from the Ethics Committee of the University of Bologna (protocol no. 0032007, dated 6 February 2024). Authorization to conduct the study in the hospital facilities was granted by the respective hospital directors.

## 3. Results

### 3.1. Background Factors

A total of 46 nurses completed the questionnaire which corresponds to approximately 50% of the nursing staff assigned to pediatric inpatient wards. However, the number of professionals who responded was lower than the value estimated by the Sample Size Calculator, which—based on a 95% confidence level, a 5-point confidence interval, and the size of the target population—indicated a required sample size that exceeded the actual number of respondents by 29. Of these, 84.8% were female and 15.2% male. Among the respondents, 17% reported having children, and 50% had experienced hospitalization with their own children.

The mean age of the participants was 40.6 years (SD = 10.7). The age distribution was as follows: 32.6% were between 21 and 24 years old, another 32.6% were between 25 and 46 years old, and 34.8% were between 47 and 57 years old.

Regarding educational background, 40% were generalist nurses, 10.9% held a master’s degree in pediatric nursing, and 2.2% had a specialist degree.

Participants had an average of 17.1 years (SD = 11.4) of professional experience and 10.8 years (SD = 9.72) of experience working in pediatric clinical settings.

Notably, 63% of respondents reported working in an organizational environment where collaboration with other healthcare professionals (e.g., physicians, physiotherapists, or speech therapists) was described as fluent (Table 1).

Overall, Table 2 shows good internal consistency of the instrument (α = 0.958), with Cronbach’s alpha ranging from α = 0.923 for cognitive-behavioral methods to α = 0.700 for creating a comfortable environment. The KMO indicates a substantially adequate sample size for all variables and for the instrument as a whole.

### 3.2. Use of Non-Pharmacological Methods by Nurses

Section two of the questionnaire consisted of a list of items asking nurses to indicate how frequently they used specific non-pharmacological methods to relieve postoperative pain in children. The reported frequencies of these interventions are summarized in Table 3 [18]. The table presents nurses’ use of cognitive-behavioral techniques, physical methods, emotional support, assistance with daily activities, and efforts to create a comfortable environment, comparing their application in relation to children and parents.

Preparatory information was explored through several questions, detailed in Table 4, including topics discussed before the procedure and sensory information. The use of these elements was compared between parents and children. In addition, the study explored which non-pharmacological techniques nurses used to provide preoperative information, such as tools employed, types of imagery, distraction strategies, and modes of information delivery.

With regard to children, the most frequently performed actions—reported as “almost always” or “always”—were the provision of preparatory information (89.1%), distraction (84.8%), guided imagery (78.3%), positive reinforcement (71.8%), and relaxation techniques (69.6%). As shown in Table 4, the most commonly used imagery techniques included recalling a favorite activity (91.3%), remembering a pleasant place (80.4%), or evoking memories of a trip or excursion (73.9%). Regarding distraction strategies, nurses most frequently talked about the child’s everyday life (91.3%), used toys during care (87%), allowed the child to watch television or videos (84.8%), played music (69.6%), and used humor (67.4%). Less frequently employed techniques included reading books, arts, and hobbies, which were all used in fewer than 60% of cases.

When comparing the interventions performed on children with those used to educate parents using the same cognitive-behavioral methods, a significant difference was observed only for breathing techniques. Nurses reported applying breathing techniques to children in 67.4% of cases, while they educated parents on this method in 89.1% of cases (*p* = 0.018, Table 3).

As shown in Table 3 and Table 4, both children and parents were well informed about surgical procedures, with 89.1% of nurses stating they provided such information to each group. However, across 12 specific preoperative information items, children received less information than parents. For instance, 63% of children were informed about the type of anesthesia and postoperative positioning, while 87% received general preoperative instructions such as fasting and premedication. Statistically significant differences between children and parents were observed in the following areas: information on the duration of the surgery was provided in 54.3% of children compared to 84.8% of parents (*p* = 0.007); information on the type of anesthesia was given to 63% of children and 91.3% of parents (*p* = 0.004); postoperative limitations were discussed with 78.3% of children and 95.7% of parents (*p* = 0.046); and information on pain medications was provided to 76.1% of children and 95.7% of parents (*p* = 0.026).

Sensory information, including aspects such as pain and nausea, was collected after the procedure in 78.3% of children and 82.6% of parents. These values were higher than those related to the assessment of sensations before the procedure (such as fear and anxiety), which were collected in 71.7% of both groups. During the procedure, sensory information was collected in 63% of children and 80.4% of parents.

The tools described in the questionnaire were generally underutilized with children. Nurses reported “almost always” or “always” using demonstrations in 34.8% of cases, books or instructional folders in 30.4%, videos in 19.6%, and preoperative surgical ward visits in only 17.4% of cases.

### 3.3. Physical Methods

Among the physical methods applied “almost always/always” to children, positioning was most commonly used (78.3%), followed by thermal regulation (57.6%), massage (32.6%), and TENS (transcutaneous electrical nerve stimulation) with only 10.9%. Similar frequencies were observed in educational actions directed toward parents on these methods, with the only significant difference recorded for TENS (*p* = 0.021):

While only 10.9% of nurses reported using TENS with children, 32.6% reported they “almost always/always” explained TENS to parents (Table 3).

### 3.4. Emotional Support

No significant differences were found between the group of interventions directed at children and those used to educate parents. Touch was the most frequently used emotional support technique (“almost always/always” in 84.8% of cases), followed by comfort and reassurance (82.6%) and nurse presence (56.5%).

No significant differences emerged between the two groups. Assistance with daily activities was reported in 67.4% of cases and creating a comfortable environment in 82.6% of interventions directed at children (Table 3).

The methods used to provide information to children were reported as being adapted “almost always/always” to the child’s age and developmental stage in 97.8% of cases. Nurses also encouraged children to speak openly about their feelings (82.6%) and provided honest and transparent information (82.6%). In addition, 80.4% of nurses ensured that the information was understood, and 69.6% encouraged children to ask questions or express concerns regarding preconceived notions (Table 4) [18].

The table also shows that most nurses “almost always/always” used imagery-based non-pharmacological techniques such as imagining a pleasant place (80.4%) and recalling a favorite activity (91.3%). Conversely, tools such as videos and preoperative visits to the surgical unit were reported as being used “not at all/very rarely” in 65.2% and 69.6% of cases, respectively, during the preoperative phase.

In Table 5, the median values appear to be generally satisfactory. This section included descriptive questions based on a four-point Likert scale (1–4), ranging from “not at all” and “a little” to “quite a bit” and “very much.” A median value of 3 was observed for most outcomes perceived by nurses regarding the effectiveness of their educational interventions toward parents, as well as their own training on pain management, non-pharmacological techniques (NPTs), and the applicability of such strategies.

Although nurses reported that NPTs are highly effective, they also stated that, during their shifts, they often have limited time to implement them.

### 3.5. Background Factors Related to the Use of Non-Pharmacological Methods

Table 6 presents the total mean Likert scores of the instrument’s variables in relation to the sample characteristics. No statistically significant associations were found between the sample characteristics (age, education, years of experience, and hospital facility) and the instrument dimensions. However, significant differences emerged in relation to an organizational factor, specifically the level of fluency in interprofessional cooperation. In pediatric wards, when interprofessional collaboration was fluent, significantly higher scores were recorded in “Providing emotional support” (12.8 ± 1.8; *p* = 0.02) and “Parental guidance” (148 ± 17.2; *p* = 0.01). Multiple comparisons using Tukey’s HSD test confirmed the ANOVA results, showing significant mean differences (MDs). Fluent interprofessional collaboration showed a significant difference compared to moderate collaboration in “Cognitive-behavioral methods” (MD = 40.7; *p* = 0.04). Moreover, fluent collaboration compared to poor collaboration was associated with significant differences in “Cognitive-behavioral methods” (MD = 53.7; *p* < 0.01), “Emotional support” (MD = 4.26; *p* = 0.02), and “Parental guidance” (MD = 35.6; *p* = 0.01).

## 4. Discussion

Overall, this study demonstrated that nurses do make use of NPTs in clinical practice. Compared to the original study, no statistically significant associations were found between background factors and the use of NPTs. However, significant findings emerged in relation to the use of NPTs to provide preoperative information to children. Notably, reading books, engaging in art, and promoting hobbies were used in fewer than 60% of cases. The use of books or comics to explain pain, particularly in the preoperative phase, is known to be particularly effective. Indeed, some literature reviews suggest the use of illustrated books and comics among NPT practices, as these simple interventions have proven effective in relieving both physical and emotional pain in children [19]. In the study, the percentage data on the use of such simple practices may be explained by the excessive nursing workload [20], understaffing [18], and the limited time available to professionals due to their commitment to performing time-consuming care procedures [21]. However, the study highlights the significant use of audiovisual devices. The literature agrees that cartoons featuring pleasant content are an effective focus of attention for most children and appear to be a safe and easy-to-administer stimulus for distraction therapy [22,23]. In fact, it has been shown that a passive strategy (such as watching TV) may be more effective than an active one (such as distraction with an interactive toy) in reducing pain, as this approach appears to lessen the child’s discomfort related to their ability to interact with the distractor [21].

A high proportion of pediatric patients undergoing surgical procedures experience acute postoperative pain. To prevent this, several interventions can be implemented. Experts recommend that adequate information be provided to both the patient and their family to ensure optimal postoperative pain management. Preoperative educational interventions should be personalized to the individual patient [24,25,26].

The data also showed that massage and TENS were rarely used by nurses in the postoperative period.

A systematic review aimed at assessing the efficacy, acceptability, and sustainability of non-pharmacological pain management interventions for older adults in China demonstrated the effectiveness of non-pharmacological techniques. The research of the articles was conducted on six databases; this study involved 2197 participants with a mean age of 69.19 years. Non-pharmacological interventions evaluated included psychotherapy, acupuncture, exercise, massage, neurotherapy, and multidisciplinary interventions. Non-pharmacological interventions have been effective in alleviating pain intensity among older adults. This evidence supports the use of these techniques even in children [27].

A systematic review conducted in accordance with the Preferred Reporting Items for Systematic Reviews and Meta-Analyses (PRISMA) guidelines analyzed several studies on NPTs for pain relief in children undergoing surgery, including 11 scientific articles published between 2019 and 2022. Cognitive-behavioral techniques were the most commonly used, while physical methods were used less frequently. The most widely adopted NPTs included music therapy, videos, and therapeutic play. Nurses can integrate these NPTs into their daily clinical practice, as their promising role in reducing pediatric postoperative pain is supported by growing evidence. Education and practical training in the use of these therapies are essential to ensure correct administration [17,28]. Nurses play a key role in managing these techniques, not only in their application but also in promoting and evaluating their effectiveness. This must be achieved through a multimodal approach tailored to the individual characteristics and clinical needs of the child [29]. A crucial aspect that emerged from this study is nurses’ perception of their own training and the time available during shifts to apply NPTs. Despite a general belief in their effectiveness, the perceived lack of time to implement these interventions during the work shift represents a significant barrier [30,31,32,33].

A study conducted in 2023 aimed to assess the use of and barriers to non-pharmacological pain management by nurses in intensive care units. A cross-sectional descriptive quantitative design was used to collect responses from a sample of 215 healthcare professionals. This study was based on three tool packages. The first part concerns demographic data. The second is a tool that uses a 4-point Likert scale to examine nurses’ use of non-pharmacological methods for pain treatment in intensive care and consists of 16 items. The third part includes six items related to perceived barriers to the use of non-pharmacological methods for pain treatment. The three main perceived obstacles to the use of non-pharmacological methods were lack of time, workload, and patient instability, reported by 83.7% (n = 180) and 77.2% (n = 166), respectively. Demographic variables were not found to be significantly associated with the use of non-pharmacological methods for pain management, except for age. The conclusion emphasized the importance of making a concerted effort to overcome the obstacles posed by lack of time and workload in order to increase the clinical use of non-pharmacological methods for pain treatment [20].

The use of a shared language to assess pain, along with the implementation of non-pharmacological methods to reduce symptom intensity and associated anxiety, forms the basis for proper care in pediatric patients [34]. The presence of at least one parent has also been shown to be a key element in ensuring the effectiveness of non-pharmacological interventions in pediatric settings [35,36,37].

Finally, the analysis showed that fluent multidisciplinary collaboration within hospital units was associated with better preoperative preparation of children. Collaboration is an active process that requires perseverance, effort, personal motivation, education, and information exchange; all of which can be difficult to achieve given the daily pressures and routines of clinical practice. A study conducted in Italy in pediatric hematology-oncology had the main objectives of improving and promoting technical and professional integration between nurses and doctors. The project was based on the theory of Appreciative Inquiry (AI), involving various European contexts. This project emphasized the importance of bringing together doctors and nurses to learn together. The authors believe that the same approach could be used to improve multiprofessional work in the care of other childhood illnesses [38,39,40].

This suggests that investing in a culture of interprofessional cooperation could further improve the quality of pediatric care [31,32,33].

## 5. Limitations

The main limitations of this study are the small sample size and its multicenter design.

Future research should aim to involve specialized pediatric surgical centers across the country and conduct a multicenter investigation to generate more robust data on the topic. Moreover, our findings highlight multidisciplinary collaboration as a positive factor in the successful implementation of NPTs. Future studies should consider involving other healthcare professionals to provide a more comprehensive understanding of how NPTs are used in pediatric pain management.

## 6. Conclusions

This observational study highlighted the importance of using NPTs, providing an overall picture of their application in the management of pediatric pain among school-aged children undergoing surgery in hospital facilities in the provinces of Ravenna, Forlì-Cesena, and Rimini. These findings offer valuable insights for future research and the further development of NPTs in pediatric settings. However, a significant communication gap emerged between children and their parents, underlining the need to improve direct dialog with children in order to ensure a less traumatic surgical experience. Adequate preparatory information can help reduce anxiety and improve postoperative outcomes, as demonstrated in previous research. Multidisciplinary collaboration also emerged as a key element in delivering high-quality care. The promotion of innovative strategies for pain education and the implementation of NPTs is essential for effective pediatric pain management. These approaches not only improve the quality of life for children and their families but also contribute to the training of competent healthcare professionals.

## Figures and Tables

**Table 1:** Sample Characteristics.

	n (%)
Gender	
Female	39 (84.8)
Male	7 (15.2)
Age group (years)	
21–24	15 (32.6)
25–46	15 (32.6)
47–57	16 (34.8)
Education	
Bachelor’s degree in nursing	40 (87.0)
Master’s degree in nursing	5 (10.9)
Specialist/master’s degree	1 (2.2)
Years of professional experience	
0–5	7 (15.2)
6–10	12 (26.1)
>10 years	27 (58.7)
Years of experience in pediatric settings	
0–5	19 (41.3)
6–10	10 (21.7)
>10 years	17 (37.0)
Hospital facility assignment	
Ravenna, Faenza, and Lugo	22 (47.8)
Cesena and Forlì	18 (39.1)
Rimini	6 (13.0)
Children	
No children	17 (37.0)
Children	29 (63.0)
Children’s previous hospitalizations	
No hospitalizations	23 (50.0)
At least one hospitalization	23 (50.0)
Fluency of interprofessional collaboration	
Poor	2 (4.3)
Moderate	15 (32.6)
Fluent	29 (63.0)

**Table 2:** Internal consistency of the survey instrument.

	Alpha	KMO
Cognitive-behavioral methods	0.923	0.759
Physical methods	0.820	0.907
Emotional support	0.802	0.784
Assistance with daily activities	0.732	0.786
Creating a comfortable environment	0.700	0.833
Parental guidance	0.916	0.755
Total	0.958	0.794

**Table 3:** Nurses’ use of non-pharmacological methods: comparison between interventions directed at children and educational interventions for parents.

	Interventions Directed at the Child			Educational Interventions for the Parent				
	Not at All/Very Rarely	Sometimes	Almost Always/Always	Not at All/Very Rarely	Sometimes	Almost Always/Always		
	n (%)						X^2^	*p*
Cognitive-Behavioral Methods								
Preoperative information	--	5 (10.9)	41 (89.1)	--	5 (10.9)	41 (89.1)	--	--
Guided imagery	1 (2.2)	9 (19.6)	36 (78.3)	2 (4.3)	5 (10.9)	39 (84.8)	1.60	0.450
Distraction	1 (2.2)	6 (13.0)	39 (84.8)	--	5 (10.9)	41 (89.1)	1.14	0.565
Relaxation	3 (6.5)	11 (23.9)	32 (69.6)	2 (4.3)	4 (8.7)	40 (87.0)	4.36	0.113
Breathing techniques	5 (10.9)	10 (21.7)	31 (67.4)	--	5 (10.9)	41 (89.1)	8.06	0.018 *
Positive reinforcement	6 (6.5)	20 (21.7)	66 (71.8)	1 (2.2)	8 (17.4)	37 (80.4)	0.60	0.741
Physical Methods								
Thermal regulation	16 (17.4)	23 (25.0)	53 (57.6)	2 (4.3)	13 (28.3)	31 (67.4)	2.19	0.334
Massage	12 (26.1)	19 (41.3)	15 (32.6)	8 (17.4)	12 (26.1)	26 (56.5)	5.33	0.070
Positioning	2 (4.3)	8 (17.4)	36 (78.3)	1 (2.2)	7 (15.2)	38 (82.6)	0.45	0.797
TENS	38 (82.6)	3 (6.5)	5 (10.9)	26 (56.5)	5 (10.9)	15 (32.6)	7.75	0.021 *
Emotional Support								
Presence	5 (10.9)	15 (32.6)	26 (56.5)	4 (8.7)	6 (13.0)	36 (78.3)	5.58	0.061
Comfort/reassurance	1 (2.2)	7 (15.2)	38 (82.6)	1 (2.2)	4 (8.7)	41 (89.1)	0.932	0.627
Touch	4 (8.7)	3 (6.5)	39 (84.8)	3 (6.5)	3 (6.5)	40 (87.0)	0.15	0.925
Assistance with daily activities	5 (10.9)	10 (21.7)	31 (67.4)	2 (4.3)	4 (8.7)	40 (87.0)	5.00	0.082
Creating a comfortable Environment	2 (4.3)	6 (13.0)	38 (82.6)	1 (2.2)	4 (8.7)	41 (89.1)	0.84	0.655

TENS = Transcutaneous Electrical Nerve Stimulation. * Statistical significant.

**Table 4:** Preoperative information and use of Non-Pharmacological techniques.

	Interventions Directed at the Child			Educational Interventions for the Parent				
	Not at All/Very Rarely	Sometimes	Almost Always/Always	Not at All/Very Rarely	Sometimes	Almost Always/Always		
	n (%)						X^2^	*p*
1. Cognitive and Sensory Information								
Topics discussed before the procedure								
15.1 What type of procedure will be performed	7 (15.2)	9 (19.6)	30 (65.2)	1 (2.2)	5 (10.9)	40 (87.0)	7.07	0.029 *
15.2 Where the procedure will take place	2 (4.3)	8 (17.4)	36 (78.3)	1 (2.2)	5 (10.9)	40 (87.0)	1.24	0.539
15.3 Who will perform the procedure	6 (13.0)	8 (17.45)	32 (69.6)	4 (8.7)	5 (10.9)	37 (80.4)	1.45	0.483
15.4 Why the procedure is important	5 (10.9)	8 (17.4)	33 (71.7)	2 (4.3)	2 (4.3)	42 (91.3)	5.97	0.051
15.5 How long the procedure will last	9 (19.6)	12 (26.1)	25 (54.3)	3 (6.5)	4 (8.7)	39 (84.8)	10.1	0.007 **
15.6 Preparations for the procedure (e.g., fasting, premedication, etc.)	1 (2.2)	5 (10.9)	40 (87.0)	--	1 (2.2)	45 (97.8)	3.96	0.138
15.7 Type of anesthesia (general/local)	9 (19.6)	8 (17.4)	29 (63.0)	1 (2.2)	3 (6.5)	42 (91.3)	11.1	0.004 **
15.8 Postoperative positioning (e.g., recovery room, inpatient ward, intensive care unit)	8 (17.4)	9 (19.6)	29 (63.0)	2 (4.3)	6 (13.0)	38 (82.6)	5.61	0.067
15.9 Postoperative monitoring in the ward	4 (8.7)	6 (13.0)	36 (78.3)	1 (2.2)	2 (4.3)	43 (93.5)	4.42	0.110
15.10 Postoperative limitations (e.g., which activities the child may or may not perform)	4 (8.7)	6 (13.0)	36 (78.3)	1 (2.2)	1 (2.2)	44 (95.7)	6.17	0.046 *
15.11 Pain medications after the procedure	4 (8.7)	7 (15.2)	35 (76.1)	1 (2.2)	1 (2.2)	44 (95.7)	7.33	0.026 *
15.12 Other pain relief methods	5 (10.9)	5 (10.9)	36 (78.3)	--	4 (8.7)	42 (91.3)	5.57	0.062
Sensory Information								
20.1 Sensations before the procedure (e.g., fear, anxiety)	3 (6.5)	10 (21.7)	33 (71.7)	2 (4.3)	11 (23.9)	33 (71.7)	0.148	0.884
20.2 Sensations during the procedure (e.g., pain)	7 (15.2)	10 (21.7)	29 (63.0)	2 (4.3)	7 (15.2)	37 (80.4)	4.28	0.118
20.3 Sensations after the procedure (e.g., pain, nausea)	1 (2.2)	9 (19.6)	36 (78.3)	2 (4.3)	6 (13.0)	38 (82.6)	0.987	0.610
Tools Used								
18.1 Books/instruction folders	18 (39.1)	14 (30.4)	14 (30.4)					
18.2 Videos	30 (65.2)	7 (15.2)	9 (19.6)					
18.3 Demonstrations	24 (52.2)	6 (13.0)	16 (34.8)					
18.4 Preoperative visit to the surgical ward with the child	32 (69.6)	6 (13.0)	8 (17.4)					
Types of imagery used								
27.1 A pleasant place	2 (4.3)	7 (15.2)	37 (80.4)					
27.2 A nice excursion or trip	6 (13.0)	6 (13.0)	34 (73.9)					
27.3 A favorite activity	--	4 (8.7)	42 (91.3)					
Types of distraction used								
30.1 Books/magazines	10 (21.7)	9 (19.6)	27 (58.7)					
30.2 Talking about daily life	1 (2.2)	3 (6.5)	42 (91.3)					
30.3 Playing with toys	3 (6.5)	3 (6.5)	40 (87.0)					
30.4 Watching television/videos	2 (4.3)	5 (10.9)	39 (84.8)					
30.5 Listening to music	4 (8.7)	10 (21.7)	32 (69.6)					
30.6 Arts/hobbies	8 (17.4)	12 (26.1)	26 (56.5)					
30.7 Humor	4 (8.7)	11 (23.9)	31 (67.4)					
2. Ways of providing information								
Encouraging the child to ask questions or share concerns	4 (8.7)	10 (21.7)	32 (69.6)					
Providing honest and open information	1 (2.2)	7 (15.2)	38 (82.6)					
Ensuring that the information is understood	1 (2.2)	8 (17.4)	37 (80.4)					
Taking into account the child’s age and developmental level	--	1 (2.2)	45 (97.8)					
Providing more detailed information to school-aged children	4 (8.7)	9 (19.6)	33 (71.7)					
Talking openly about the child’s feelings and sensations	--	8 (17.4)	38 (82.6)					

* Statistical significant.

**Table 5:** Nurses’ perceptions of educational outcomes for parents and their own training.

	Median	Mode	M ± SD
Educational Outcomes			
Do you generally believe that parents adequately prepare their child for surgery?	3.00	3.00	2.80 ± 0.83
Nurse Training			
Do you believe your training in pediatric pain management is adequate?	3.00	3.00	3.22 ± 0.72
Do you believe your training in NPTs is adequate?	3.00	3.00	2.98 ± 0.88
Do you feel that you have enough time during your shift to apply NPTs?	2.00	2.00	2.28 ± 1.00
Do you find it easy to apply NPTs in children with disabilities?	3.00	3.00	2.59 ± 0.97
Perceived Outcomes of NPTs			
Finally, do you consider NPTs to be effective?	4.00	4.00	3.41 ± 0.71

NPTs = non-pharmacological techniques.

**Table 6:** Differences between the sample variables and the instrument areas.

	Cognitive-Behavioral Methods	Emotional Support	Parental Guidance
Range	42–210	3–15	33–165
M ± DS (I.C.)			
Fluency of interprofessional collaboration			
Poor	118.0 ± 2.8 (92.6–143.4)	8.5 ± 0.7 (2.2–14.9)	112.5 ± 6.3 (22.3–169.7)
Moderate	158.7 ± 18.9 (148.3–169.1)	11.7 ± 2.8 (10.2–13.3)	140.2 ± 16.8 (130.9–149.5)
Fluent	171.7 ± 23.6 (162.7–180.7)	12.8 ± 1.8 (12.1–13.5)	148.1 ± 17.2 (141.6–154.7)
	F = 6.56, *p* = <0.01	F = 4.24, *p* = 0.02 *	F = 4.70, *p* = 0.01 *

* Statistical significant.

## Data Availability

Data are available from the first author upon reasonable request.

## Public Involvement Statement

No public involvement in any aspect of this research.

## Guidelines and Standards Statement

This manuscript was drafted against the STROBE (Strengthening the Reporting of Observational Studies in Epidemiology) guidelines for observational research.
